# Salamander–*Batrachochytrium salamandrivorans* Interactions Through Dual Transcriptomics

**DOI:** 10.1002/ece3.73967

**Published:** 2026-07-03

**Authors:** María Torres‐Sánchez

**Affiliations:** ^1^ Department of Life, Health, and Environmental Sciences University of L'Aquila L'Aquila Italy; ^2^ Department of Biodiversity, Ecology, and Evolution Complutense University of Madrid Madrid Spain; ^3^ Department of Biology University of Florida Gainesville Florida USA

**Keywords:** *Batrachochytrium dendrobatidis*, *Batrachochytrium salamandrivorans*, co‐infection, *Desmognathus*, dual RNA‐seq, *Eurycea*, *Notophthalmus*, *Tylototriton*

## Abstract

Changes in gene expression during host–pathogen interactions reveal species' functional responses and provide insight into the mechanisms by which pathogens impact biodiversity. The pathogen *Batrachochytrium salamandrivorans* (Bsal) poses a major threat to salamander diversity, particularly in global biodiversity hotspots. In the most species‐rich salamander family, Plethodontidae, infection outcomes vary markedly, suggesting that hosts play a role in mediating susceptibility. Despite this variation, salamander defence mechanisms against Bsal, as well as the pathogen's capacity to facilitate multi‐host infection, remain largely unexplored. Here, I characterised gene expression changes associated with salamander–Bsal interactions using a multispecies comparative framework that integrated host and pathogen functional mechanisms. I identified variation in host responses alongside a conserved expression pattern among plethodontid salamanders, and uncovered Bsal's capacity to adjust its genetic machinery in relation to host susceptibility and, potentially, co‐infection with its sister taxon, *Batrachochytrium dendrobatidis* (Bd). These findings provide insight into the biological mechanisms underlying salamander–Bsal interactions, highlighting a pathway involved in Bsal virulence mediated by calmodulin. This study underscores the need to characterise gene expression changes in both host and pathogen simultaneously to better understand species interactions, and proposes that these changes should be modelled within the parasite–mutualist continuum. Comparative frameworks incorporating multiple host species are also essential for characterising conserved responses to infection, and ultimately for anticipating, preventing and mitigating the potential negative impacts of Bsal on salamander biodiversity.

## Graphical abstract

Using dual transcriptomics, this study reveals how salamanders defend against an emerging pathogen and how the pathogen infects multiple hosts, uncovering a novel calmodulin‐mediated virulence pathway. These mechanistic insights connect molecular responses to host susceptibility, advancing our understanding of the potential impacts of disease on salamander biodiversity hotspots.

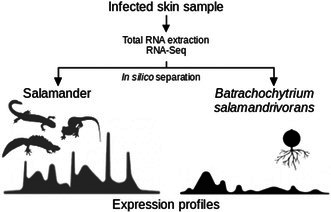

## 1 Introduction

Coding and non‐coding RNAs are probably the most informative molecules for revealing how species respond to ecological interactions. Organisms interact continuously among themselves and with their environment and react to changes through a myriad of biological processes, which usually require the activation of differential genetic machinery. Hence, studying changes in gene expression associated with different biological relationships, such as host–pathogen interactions, can enable the characterisation of the species response to those liaisons. In this rapidly changing world, a better understanding of species responses to biological interactions could help to prevent and mitigate the negative impacts of ecosystem alterations on biodiversity (Theissinger et al. 2023).

One of the most catastrophic ecosystem changes is the introduction of invasive species that can result in the emergence of new infectious diseases (Zhang et al. 2022). In the last decades, human activities, movement and globalisation have blurred the geographical barriers and the dispersal limits of many species. Among them are the *Batrachochytrium* fungi (*B. dendrobatidis* or Bd and *B. salamandrivorans* or Bsal), which are the etiological agents of the amphibian skin infectious disease known as chytridiomycosis (Longcore et al. 1999; Martel et al. 2013). This disease is one of the reasons for the ongoing decline of amphibians, which are considered the most endangered vertebrate group (Fisher and Garner 2020; Luedtke et al. 2023; Scheele et al. 2019). The native range of both fungal species is presumably located in Asia (Martel et al. 2014; O'Hanlon et al. 2018). Bd affects all three amphibian orders, more severely anurans and is globally widespread, whereas Bsal mainly impacts urodeles with an invasion range encompassing several European countries at the present time (Martel et al. 2014; Spitzen‐van der Sluijs et al. 2016). The potential invasion risk of Bsal has spurred a broad diversity of research to assess and prevent the catastrophic consequences of this pathogen in salamander biodiversity hotspots (see for example Basanta et al. 2019; Bosch et al. 2021; Gray et al. 2023).

Eastern North America harbours a great salamander diversity, being the region where the largest family of the group (Plethodontidae Gray, 1850) reaches its highest local species richness. This diverse and unique group of lungless salamanders has experienced population declines mainly associated with habitat loss and climate change (Luedtke et al. 2023). The already imperilled status of some plethodontids could worsen due to the expected synergistic effects of Bsal, especially in species previously described as susceptible (Carter et al. 2020; DiRenzo et al. 2021; Friday et al. 2020). This is the case of *Desmognathus auriculatus* (Holbrook, 1838), commonly known as the southern dusky salamander, whose populations are suffering severe declines and local extinctions (Graham et al. 2010; Means and Travis 2007). Additionally, this dusky salamander is highly susceptible to both Bd, which is present in its distribution range, and Bsal, currently absent in America (Friday et al. 2020). Other species of the same genus inhabiting the same region appear potentially better able to keep pace with anthropogenic pressures, showing no susceptibility to Bsal (e.g., *D. apalachicola* Means and Karlin, 1989) (Friday et al. 2020). The outcome of Bsal infection is quite variable across the Plethodontidae tree of life and little is known about the genetic machinery involved in fighting the infection (DiRenzo et al. 2021; Gray et al. 2023). Only two previous studies have independently characterised gene expression changes associated to Bsal infection in two susceptible species of the family Salamandridae Goldfuss, 1820 (Farrer et al. 2017; McDonald et al. 2020).

**FIGURE 1:**
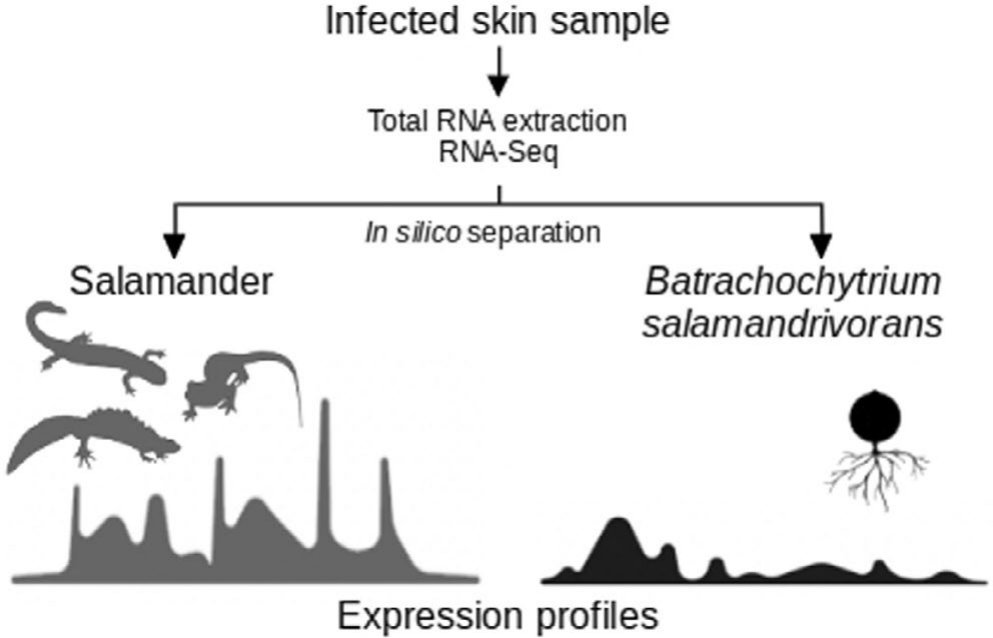
Schematic representation of the dual transcriptomics approach used to study salamander—*Batrachochytrium salamandrivorans* interactions. For Bsal‐infected skin samples, RNA was extracted, sequenced and reads were separated by organism through mapping to the Bsal reference genome and reconstructing de novo assemblies, allowing analysis of gene expression profiles for both the salamander host and Bsal (silhouette images from PhyloPic).

In this study, I explored gene expression changes associated with salamander–Bsal interactions using a multispecies comparative framework, analysing both previously published and newly generated transcriptomes (Figure 1). I aimed to address two questions: (i) whether plethodontids as naïve hosts exhibit a conserved gene expression response to Bsal infection; and (ii) whether Bsal displays different gene expression profiles across host environments, including host type (susceptible vs. non‐susceptible) and Bd competition (single vs. co‐infection). I found variation in the host responses alongside a conserved expression pattern in plethodontid salamanders, and uncovered Bsal's capacity to adjust its gene expression in relation to host susceptibility and, potentially, co‐infection with Bd.

## 2 Materials and Methods

### 2.1 Infection Experiment and Gene Expression in the Skin and Spleen of Five Plethodontidae Species

Individuals from five lungless salamanders (four individuals per species) were selected for this study. These animals were part of a Bsal infection experiment previously conducted by Friday et al. (2020) to assess the pathogen's impact on naïve species. Individuals were wild‐caught, and natural subclinical Bd infections were detected in four of the five species (*D. apalachicola, D. conanti* Rossman, 1958, *Eurycea cirrigera* [Green, 1831] and *E. guttolineata* [Holbrook, 1838]), with the exception of *D. auriculatus* . Salamanders were untreated and randomly exposed to 10^4^ Bsal zoospores and to sham solutions in treatment and control categories, respectively, for 24 h. After the infection experiment, individuals were euthanised between 3 and 7 weeks post‐exposure (timing determined by mortality progression in each species; see Friday et al. 2020 for further details) and fresh skin and spleen tissues were collected from each species and experimental category and stored in RNAlater at −80°C until purification. I homogenised tissue samples using TissueRuptor II for subsequent RNA extraction with QIAGEN RNeasy Plus Mini Kit, and tested RNA quantity and quality through Qubit and Bioanalyzer assays. I prepared cDNA libraries using NEBNext Poly(A) mRNA Magnetic Isolation Module and NEBNext Ultra II Directional RNA Library Prep Kit following manufacturers' protocols. The 20 libraries (five from Bsal‐infected skins, five from control skins, five from Bsal‐infected spleens and five from control spleens, one per each plethodontid species) were pooled in equimolar ratios to short‐sequence (paired‐end 150 reads) on an Illumina NovaSeq 6000 S4 flow cell (see Table S1 for further information about the newly generated transcriptomes including the NCBI SRA ids of the samples).

I checked transcriptome read quality using FastQC 0.11.5 (https://github.com/s‐andrews/FastQC) and subsequently filtered Illumina Universal Adapters and trimmed the 10 left‐bases of the reads using Trim Galore 0.6.5 (https://github.com/FelixKrueger/TrimGalore) and Prinseq 0.20.4 (Cantu et al. 2019). I also removed reads that mapped against the pathogen (see Section 2.2 for more information about Bsal gene expression analyses). Following a multiple assemblers and multiple parameters strategy, I performed de novo transcriptome assemblies for each of the salamander species using all their samples (four samples for each species: two skins and two spleens from control and infected animals, respectively: one Bsal‐infected skin, one control skin, one Bsal‐infected spleen and one control spleen). I first normalised the data applying the in silico read normalisation of Trinity 2.11.0. (Haas et al. 2013). Using the normalised reads, I reconstructed transcripts with different k‐mer sizes and assemblers, namely Trinity 2.11.0 (Haas et al. 2013), SPAdes 3.15.0 (Bushmanova et al. 2019), SOAPdenovo‐trans 1.03 (Xie et al. 2014), TransABySS 2.0.1 (Robertson et al. 2010) and Velvet 1.2.10 (Zerbino and Birney 2008) in combination with Oases 0.2.08 (Schulz et al. 2012). I built a total of 20 de novo assemblies for each species. After concatenating the assemblies of each salamander, I selected the coding sequences (CDS) with more than 200 nucleotides from the overassembly files with tr2aacds of EviGene 2020.05 (Gilbert 2019), and clustered output sets with CD‐HIT 4.6.8 using a sequence similarity cut off of 95% (Fu et al. 2012). De novo transcriptome assemblies were uploaded to the NCBI TSA database following recommended best practices for open science and green computing (Torres‐Sánchez et al. 2025).

To compare gene expression across salamander species and tissue samples, I first used Bowtie2 2.4.2 to map each filtered transcriptomic sample against its final de novo assembly (Langmead et al. 2009), counting total reads per transcript with eXpress 1.5.1 (Roberts and Pachter 2013). I used OrthoFinder 2.5.2 to identify comparable orthologous sequences across species creating a unique expression dataset using single copy orthologs (Emms and Kelly 2015). I annotated these orthologs against Uniprot using BLAST 2.9.0 while recovering gene ontology (GO) information using retrieve/ID mapping tool of Uniprot (Altschul et al. 1990; Bateman et al. 2017). I normalised count expression across samples for the Plethodontidae single copy orthologs' dataset applying the median of ratios method of the R package DESeq2 (Love et al. 2014). I computed variance stabilising transformations and performed a principal component analysis (PCA) representing samples using scatterplots and boxplots with the R package ggplot2 (Wickham 2017). I tested whether some of PCs sample loadings differed between the experimental categories using *t*‐tests (control vs. infected). To identify differential gene expression, I fitted the normalised count data to negative binomial models and computed Wald tests per gene comparing control versus infected samples. I visualised the candidate differential expressed genes (*p*‐value ≤ 0.05 instead of adjusted *p*‐values due to small sample size and large sample variation) using heatmaps with the R package pheatmap (https://github.com/raivokolde/pheatmap).

### 2.2 *Batrachochytrium salamandrivorans* Gene Expression Across Seven Salamander Species

I gathered all the transcriptomic data from Bsal‐infected amphibian skin and Bsal in culture samples publicly available to date. Besides the five Bsal‐infected skin transcriptomes generated here, two previous studies have investigated gene expression changes related to Bsal infection in two other species of salamanders: *Tylototriton wenxianensis* Fei, Ye and Yang, 1984 and *Notophthalmus viridescens* (Rafinesque, 1820) (Farrer et al. 2017; McDonald et al. 2020). The first study describes the gene expression response of the host to Bsal and Bd in single infections (three Bsal‐infected skin samples of *T. wenxianensis* and one in culture sample reanalysed here), whereas the second study includes the host response to not only single infections but also co‐infections with both *Batrachochytrium* species (four Bsal‐infected skin samples of *N. viridescens* reanalysed here; see Section 2.3 for the analysis of co‐infection samples). As in the infection trial of plethodontid species (Friday et al. 2020), salamanders were exposed to 10^4^ Bsal zoospores in the treatment groups of these two studies. *T. wenxianensis* were captive bred individuals, whereas *N. viridescens* were wild‐caught and treated to remove natural Bd infections prior the Bsal infection trial. Instead of focusing on the host in this analysis, I explored Bsal gene expression variation across different host environments (susceptible and non‐susceptible hosts) applying the dual RNA‐Seq strategy (Westermann et al. 2012) and following previous work on Bd (Torres‐Sánchez et al. 2022).

To recover Bsal gene expression, I aligned the Bsal in culture sample and the 12 transcriptome samples of Bsal‐infected skin (five newly generated transcriptomes: one for *D. apalachicola*, one for *D. auriculatus*, one for *D. conanti*, one for *E. cirrigera* , one for *E. guttolineata* and seven previously published transcriptomes: three for *T. wenxianensis* and four for *N. viridescens* ; see Table S1 for information about the NCBI SRA ids of the samples) to the Bsal reference genome (GCA_002006685.1) using gffread 0.11.8 (https://github.com/gpertea/gffread) and Star 2.7.3a (Dobin et al. 2013). I retrieved counts for all genes in the Bsal genome handling zero values by adding one unit to all genes across samples in the matrix count. As the samples were originated from different projects, technical variation could be expected. To ameliorate this, I normalised gene expression across samples applying the median of ratios method of the R package DESeq2 (Love et al. 2014) and estimating size factors using the reference genes: glyceraldehyde‐3‐phosphate dehydrogenase (GAPDH) and beta tubulin (TUB) (Verbrugghe et al. 2019). As in the previous section, I computed variance stabilising transformations, performed clustering analyses to study sample similarity, tested differences of the general expression pattern and identified differential gene expression comparing susceptible versus non‐susceptible hosts (adjusted *p*‐value ≤ 0.05; host susceptibility characterised based on mortality; see Table S2). To identify the potential function of the differentially expressed genes, I annotated Bsal genes against Uniprot using BLAST 2.9.0 while recovering GO information using retrieve/ID mapping tool of Uniprot (Altschul et al. 1990; Bateman et al. 2017). Additionally, to explore whether Bsal expression pattern was influenced by amphibian evolutionary history, I retrieved 10,000 phylogenetic trees for the seven salamander hosts from VertLife (Jetz and Pyron 2018), built a consensus tree using the R package phytools (Revell 2024), and identify topology mismatches between Bsal gene expression clusters and host species phylogeny.

### 2.3 *Batrachochytrium salamandrivorans* Gene Expression Variation in Simple Infection and Co‐Infection With *B. dendrobatidis* in a Newt Host

Using single Bsal infected and co‐infected with Bd skin samples of *N. viridescens* from a previous study (McDonald et al. 2020), I explored gene expression variation of Bsal when in competition with another pathogen. In this last analytical step of this study, I followed the same methodological steps described in the previous section. I aligned the four skin samples of *N. viridescens* co‐infected with Bd and Bsal to the Bsal genome reference and built a gene count matrix with these samples and the four Bsal‐infected skin samples (single infected) of the same species. I compared gene expression variation across these eight samples testing differential gene expression with the contrast single infection versus co‐infection.

## 3 Results

### 3.1 Plethodontidae Species Transcriptomes, Orthologous Groups and General Gene Expression Pattern

I reconstructed hundreds of thousands of CDS for the five Plethodontidae species (136,825 for *D. apalachicola*, 128,955 for *D. auriculatus*, 117,913 for *D. conanti*, 123,632 for *E. cirrigera* , 204,722 for *E. guttolineata* ; see Table S1 for information about the NCBI TSA ids of the de novo transcriptome assemblies). All the CDS were classified into sets of homologous genes known as orthologous groups that diverged from a single gene in the last common ancestor of the studied plethodontid salamanders, plausibly conserving sequence function. I found 26,648 orthologous groups with transcripts from the five species, being 11,718 single copy orthologs. For this last group, I described the general gene expression pattern of skin and spleen samples. Independently of the species and experimental category, I identified a general gene expression pattern related to tissue type (7.58% of the gene expression variation; see Figure S1). I also detected gene expression signatures related to salamander genera. 2.89% of the gene expression variation of the samples was explained by the genus: *Desmognathus* and *Eurycea*, with expression profiles clustering samples by genus: *D. apalachicola*, *D. auriculatus* and *D. conanti* grouped together, while *E. cirrigera* and *E. guttolineata* formed a separate cluster (Figure S1).

### 3.2 Host Gene Expression Changes in the Skin and Spleen due to Bsal Infection

**FIGURE 2:**
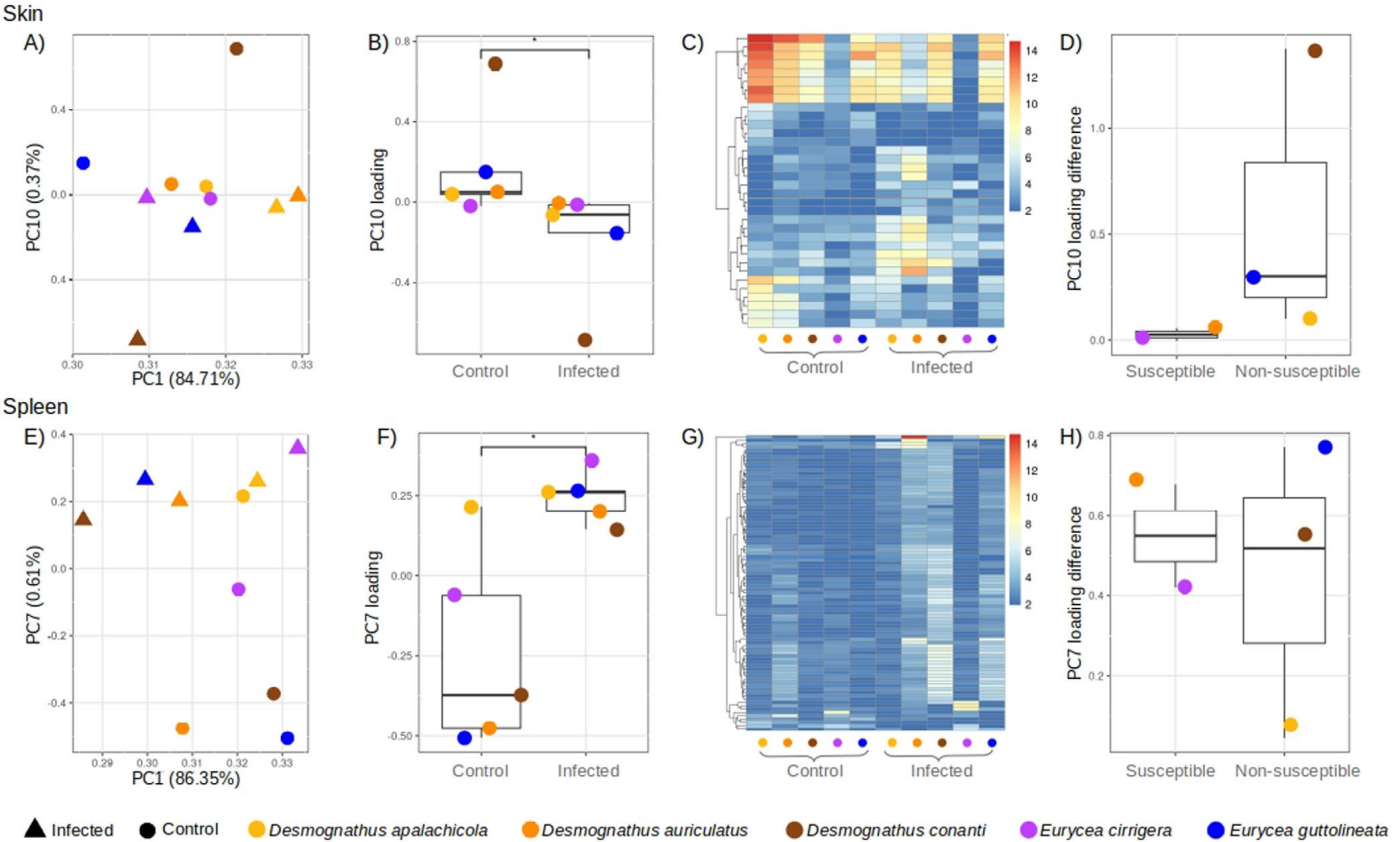
Gene expression of the plethodontid salamander hosts. General gene expression profiles and differential expression between *Batrachochytrium salamandrivorans* (Bsal) infected and control samples for the skin (A–D) and spleen (E–H). (A, E) Scatter plots representing two components of the principal component analysis (PCA) of gene expression, differentiating control and infected samples with shapes and species with colours. (B, F) Box plots of the PC10 and PC7 loadings of each sample for the skin and spleen, respectively, grouped between control and infected and colour‐coded by species. (C, G) Heat maps showing differentially expressed genes between infected and control samples. (D, H) Box plots displaying PC10 and PC7 loadings differences of each species for the skin and spleen, respectively, grouped by susceptibility.

I detected a small percentage of gene expression variation associated with infection category in both the skin and spleen samples (< 1%; see Figure 2A,E). This variation accounts for < 100 orthologs, but was overall significant between control and infected salamanders in both tissues (Figure 2B,F). I identified candidate differentially expressed genes (*p*‐value ≤ 0.05 and fold change in logarithmic scale ≥ 1) for both tissues (Figure 2C,G). A total of 34 and 84 orthologs showed differences in expression level between control and infected salamanders in the skin and spleen, respectively. In the skin, I detected more downregulation than upregulation with 19 genes reducing their expression level and 15 genes increasing their expression level in infected individuals. On the contrary, in the spleen, I found a higher number of genes that increased their expression in infected animals with 84 upregulated genes and five downregulated. Among the skin upregulated genes, I identified genes coding for proteins with binding functions to the complement component C3b, metal and calcium ions and ion channels. Several mitogen‐activated protein kinase phosphatases (MKPs) were downregulated in the skin. When comparing gene expression changes associated with host susceptibility, I found that the two susceptible species ( *D. auriculatus* and *E. cirrigera* ) showed minimal within‐species differences between control and infected skin samples (Figure 2D,H). In contrast, *D. conanti* , one of the non‐susceptible species, exhibited the largest difference between control and infected skin expression profiles.

### 3.3 Bsal Gene Expression Variation Is Associated With Host Susceptibility

**FIGURE 3:**
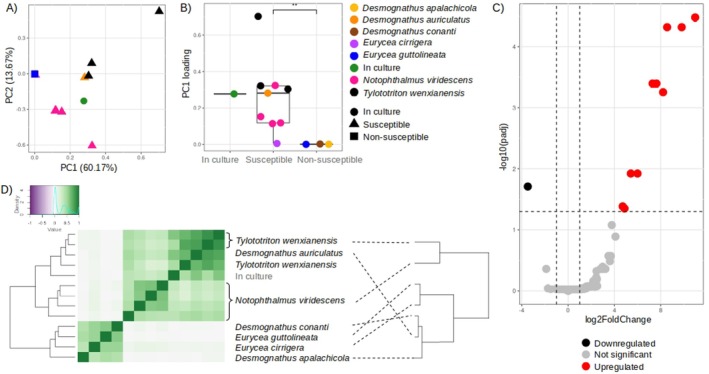
Gene expression of *Batrachochytrium salamandrivorans* (Bsal). (A) Scatter plot representing two components of the principal component analysis (PCA) of gene expression, differentiating host susceptibility with shapes and species with colours. (B) Box plot of the PC1 loadings of each sample grouped by host susceptibility and colour‐coded by species. (C) Volcano plot showing differentially expressed genes between susceptible and non‐susceptible hosts. (D) Heat map of the Bsal expression correlation across host environments paired with the phylogeny of the salamander hosts (topology to the right).

The general gene expression pattern of Bsal revealed differences across host environments (Figure 3A,D). Bsal gene expression profiles were partially explained by host susceptibility (PC1 explained 60.17%; Figure 3B). When infecting the two susceptible Salamandridae species ( *T. wenxianensis* and *N. viridescens* ), Bsal expressed a similar genetic machinery while showing variation between species and among biological replicates (Figure 3A,B,D). This general gene expression pattern was shared with the in culture sample and one of the susceptible Plethodontidae species ( *D. auriculatus* ). In the other species categorised as susceptible ( *E. cirrigera* ; see Table S2 for further details about susceptibility category), Bsal displayed a gene expression pattern more congruent with the rest of the Plethodontidae family samples (Figure 3A,D). This clustering of the Bsal gene expression revealed discrepancies with the topology of the salamander phylogeny, potentially underlying the lack of host phylogenetic signal in Bsal gene expression patterns (Figure 3D). Importantly, Bsal expression infecting *D. auriculatus* was grouped with *T. wenxianensis* samples. Besides the variation related to the general expression pattern, I identified differentially expressed genes between susceptible and non‐susceptible species samples (adjusted *p*‐value ≤ 0.05 and fold change in logarithmic scale ≥ 1; Figure 3C). I detected increased expression of 10 Bsal genes during infection of susceptible hosts, while one gene was downregulated. Most of the upregulated genes were annotated with binding functions, including those encoding heat shock proteins and calmodulin. The downregulated gene was annotated as a kelch protein.

### 3.4 Potential Competition Between Batrachochytrium Species for Amphibian Skin Resources

**FIGURE 4:**
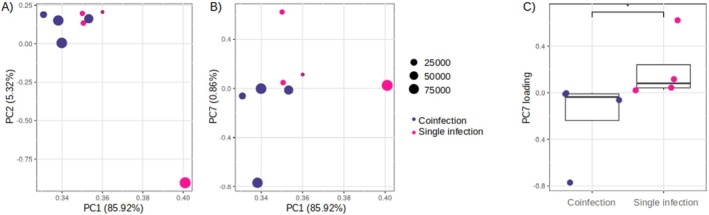
Gene expression of *Batrachochytrium salamandrivorans* (Bsal) during single and co‐infection. (A, B) Scatter plots representing two components of the principal component analysis (PCA) of gene expression, differentiating single and co‐infected samples with colours and Bsal load with size. (C) Box plot of the PC7 loadings of each sample grouped and colour‐coded by single infection and co‐infection.

When comparing Bsal gene expression during single and co‐infection in the skin of *N. viridescens* , I found an outlier related to a possible effect of Bsal load during single infection, explaining the higher percentage of variation of the general gene expression pattern (85.92%; see Figure 4A). I also detected a small percentage of Bsal gene expression variation associated with the type of infection: single or co‐infection (< 1%; see Figure 4B,C). While differences between samples at the general expression pattern were significant (Figure 4C), I had no statistical power to identify differentially expressed genes between single and co‐infected samples.

## 4 Discussion

This study provides a comparative gene expression profiling of salamander–Bsal interactions across several host species. To perform the analyses presented here, I generated transcriptomic data for five species of salamanders (*D. apalachicola*, *D. auriculatus* , *D. conanti* , *E. cirrigera* and *E. guttolineata* ) and made the assemblies publicly available, which, despite being best practice, is infrequently adopted in amphibians (Torres‐Sánchez et al. 2025). Amphibians, and within the group, especially salamanders, are less well represented by genomics resources (reference genomes or transcriptomes) than other vertebrates (Kosch et al. 2024). Moreover, amphibians are the most threatened vertebrate group (Luedtke et al. 2023). Hence, there is an urgent need to generate high‐throughput sequencing resources for conservation genomics purposes (Theissinger et al. 2023). Among the threats impacting amphibians are the emerging infectious diseases, including chytridiomycosis (Fisher and Garner 2020; Scheele et al. 2019). The different infection outcomes among species and populations of the same species puzzle researchers and ignite studies like this one. Here, I focused on the overlooked gene expression response among multiple hosts and the unexplored gene expression variation of Bsal in different host environments, establishing the first steps for a comparative host–pathogen gene expression framework. Importantly, results pointed towards a conserved expression pattern among plethodontid salamanders and uncovered Bsal's capacity to adjust its genetic machinery across host environments. These findings may prompt a rethinking of amphibian–*Batrachochytrium* species interactions through the lens of parasite–mutualist continuum (Drew et al. 2021; Ewald 1987).

To answer whether different infection outcomes are mediated by different interactions between salamanders and Bsal, I first explored the gene expression profiles of closely related hosts that present different susceptibility and mortality rates (Friday et al. 2020). The five species of plethodontids showed some similar gene expression changes, pointing to a potential conserved host response to this novel threat. It should be noted that this pattern, inferred from single‐copy genes, may not capture the broader conserved responses shared by these species. In the plethodontid skin samples, I detected an increased expression of genes annotated with binding function to C3b, a complement molecule able to attach to pathogens, signalling them for destruction (Janeway et al. 2001). This could indicate the activation of the innate immune system in plethodontids to fight Bsal. To battle infection, plethodontids could also employ a nutritional immunity strategy, restricting access to essential nutrients, such as metals, to limit pathogen growth and survival (Murdoch and Skaar 2022). Genes annotated with metal ion binding functions were upregulated in the infected skins of the studied plethodontid salamanders. These proteins could make unavailable or sequestrate essential metals for Bsal, which requires them for activating metalloproteinases to break host cells and make host resources accessible. Nutritional immunity has been proposed as a crucial process in amphibian–Bd systems in a previous study exploring Bd gene expression changes across multiple different amphibian hosts (Torres‐Sánchez et al. 2022).

Overall, I found that species from the family Plethodontidae, which are at high conservation concern due to the potential arrival of Bsal in America (Gray et al. 2023), can respond to some extent to this pathogen changing gene expression in the skin and spleen. These changes had, however, different magnitude regarding the susceptibility of the hosts. In *D. auritulatus* and *E. cirrigera*, species experiencing mortality events during a Bsal infection trial (Friday et al. 2020), I found smaller differences between control and infected samples. This pattern is congruent with previous results describing little to no gene expression change in response to Bsal infection in another susceptible salamander, *T. wenxianensis* (Farrer et al. 2017). To better characterise gene expression changes in response to Bsal, especially in relation to host susceptibility, and to support the trends described here, additional biological samples from both the same and different salamander hosts are needed, which would help to further elucidate skin and spleen gene expression patterns distinguishing local and systemic responses, respectively.

In any species interaction, there are always two or more players involved in the process. In the case of host–pathogen interactions, pathogen reaction to host response could also explain infection outcome. To determine if the pathogen can employ different infection strategies across hosts, I compared Bsal gene expression changes in the infected skin of the five plethodontids and in another two salamander hosts, for which transcriptomic resources are available (Farrer et al. 2017; McDonald et al. 2020). I detected gene expression variation in Bsal explained at some level by host susceptibility as previously shown for its sister taxon, Bd (Torres‐Sánchez et al. 2022). The virulence of Bsal could be related to a signalling process mediated by calmodulin as in other fungal pathogens (Park et al. 2019). The sequence coding for this protein was upregulated in susceptible hosts. This pathway is governed by intracellular calcium, a universal signalling molecule. Thus, any processes regulating the availability of this ion could interfere in the success of Bsal to infect a host. Previous research has shown that calcium is the signalling molecule that induces Bd zoospores to initiate sporangium development in culture (Robinson et al. 2022). Importantly, plethodontids increased the expression of genes with calcium ion binding function as part of the potential conserved machinery to fight Bsal infection. Longitudinal transcriptomic data are required to decipher sequential events during the dynamic process of infection and, hence, disentangle the cross‐kingdom communication of the amphibian–*Batrachochytrium* species interactions, which can impact the amphibian evolutionary trajectories (Torres‐Sánchez 2025).

Host environments could exert contrasting selective pressures, leading to Bsal gene expression plasticity, as shown in Bd (Torres‐Sánchez et al. 2022). The host environments explored here are novel environments for the pathogen, thus gene expression plasticity is predating potential adaptation or co‐evolution processes (with, maybe, the exception of *T. wenxianensis*, whose range might overlap with Bsal native range) (Martel et al. 2014). In this situation, closely related hosts could have, for one side, similar skin structures and components and, for the other, akin ecology, exhibiting similar host skin environments (Bletz et al. 2017; Ramírez‐Barahona et al. 2023). It could be expected that Bsal expresses the same genetic machinery when infecting species within Plethodontidae and within Salamandridae, but exhibits differences between these two families, which diverged from a common ancestor in the late Middle Jurassic (Zhang and Wake 2009). This expected pattern failed to be recovered due to the Bsal expression in *D. auriculatus* . In this plethodontid species, Bsal expressed the same general gene expression profile as in *T. wenxianensis* with a higher correlation with this species' samples and the samples from *N. viridescens* than with the rest of the plethodontid salamander samples. I hypothesised that infection outcome may play a greater role in determining the genetic machinery Bsal uses to infect different hosts, as previously described for Bd (Torres‐Sánchez et al. 2022). Yet, in the other susceptible plethodontid, *E. cirrigera* , I recovered an expression profile analogous to the other three non‐susceptible lungless salamanders. This could reflect infection progression, capturing two different infection stages. To rule out this process and other possible biological scenarios (e.g., susceptibility variation within *E. cirrigera* ), sample replicates within species are required. It should be noted that differences among the three infection trials explored here (Farrer et al. 2017; Friday et al. 2020; McDonald et al. 2020), including variation in euthanasia time points, host origin (captive‐bred vs. wild‐caught), prior Bd status (cleared vs. naturally infected), and unknown infection histories of wild‐caught individuals, may have contributed to variation in gene expression patterns of both salamander hosts and Bsal. Given Bsal's capacity to rapidly adapt and evolve (Kelly et al. 2021), characterising host environments, including the influence of other skin symbionts, such as Bd, in association with changes in Bsal gene expression profiles is necessary to better predict and mitigate Bsal risk. I therefore suggest considering this system under parasite–mutualist continuum models (Drew et al. 2021; Ewald 1987).

## 5 Conclusions

This study showed preliminary results of gene expression variation in both hosts and the pathogen during salamander–Bsal interactions. When possible, studies of host–pathogen gene expression changes should use a dual transcriptomics approach to more accurately capture this complex biological process. Assessing gene expression in both interacting species is necessary to better understand host responses to infection, and hence to prevent and mitigate the pathogen's impacts on biodiversity.

## Author Contributions

**María Torres‐Sánchez:** conceptualization (lead), data curation (lead), formal analysis (lead), investigation (lead), methodology (lead), visualization (lead), writing – original draft (lead), writing – review and editing (lead).

## Conflicts of Interest

The author declares no conflicts of interest.

## Supporting information


**Table S1:** NCBI SRA and TSA IDs. Sequencing files and de novo transcriptome assemblies are deposited in the NCBI databases under the BioProject PRJNA739374.
**Table S2:** Host species susceptibility. Category based on mortality during infection trial, if mortality events are recorded the species is considered susceptible
**Figure S1:** Skin and spleen gene expression of the plethodontid salamander hosts. General gene expression pattern for samples from five hosts (*Desmognathus apalachicola, D. auriculatus, D. conanti, Eurycea cirrigera* and *E. guttolineata* ; represented by colour), two tissues (skin and spleen; represented by shape) and different infection categories (control and infected; represented by size). Scatter plots show two components of the principal component analysis (PCA) of gene expression: (A) PC1 versus PC2; (B) PC1 versus PC4; and (C) PC2 versus PC4.

## Data Availability

Raw sequences files and transcriptome assemblies are deposited in the NCBI SRA and TSA databases (see Table S1). To ensure reproducibility, analysis code is provided as Data S1.
