# Supplementary material for: Salamander–Batrachochytrium salamandrivorans Interactions Through Dual Transcriptomics
Source: Ecol Evol. 2026 Jul 3;16(7):e73967. doi: 10.1002/ece3.73967 (PMC13329848; doi:10.1002/ece3.73967)
Supplement: Supplementary file 1 — Table S1: NCBI SRA and TSA IDs. Sequencing files and de novo transcriptome assemblies are deposited in the NCBI databases under the BioProject PRJNA739374. Table S2: Host species susceptibility. Category based on mortality during infection trial, if mortality events are recorded the species is considered susceptible Figure S1: Skin and spleen gene expression of the plethodontid salamander hosts. General gene expression pattern for samples from five hosts (Desmognathus apalachicola, D. auriculatus, D. conanti, Eurycea cirrigera and E. guttolineata ; represented by colour), two tissues (skin and spleen; represented by shape) and different infection categories (control and infected; represented by size). Scatter plots show two components of the principal component analysis (PCA) of gene expression: (A) PC1 versus PC2; (B) PC1 versus PC4; and (C) PC2 versus PC4. [file ECE3-16-e73967-s001.pdf]

SUPPLEMENTARY INFORMATION

Salamander–*Batrachochytrium salamandrivorans* interactions through dual transcriptomics

María Torres-Sánchez

Department of Life, Health, and Environmental Sciences, University of L'Aquila, 67100, L'Aquila, Italy

Department of Biodiversity, Ecology, and Evolution, Complutense University of Madrid, 28040 Madrid, Spain

Department of Biology, University of Florida, Gainesville, FL 32611, USA

Corresponding author email: María Torres-Sánchez (torressanchez.maria@gmail.com)

This file includes:

Table S1 and S2

Figure S1

Table S1. NCBI SRA and TSA IDs. Sequencing files and *de novo* transcriptome assemblies are deposited in the NCBI databases under the BioProject PRJNA739374.

Species	Tissue	Infection category	SRA	TSA
Desmognathus apalachicola	skin	control	SRR22671587	GKYX00000000
		infected	SRR22671575	
	spleen	control	SRR22671588	
		infected	SRR22671576	
Desmognathus auriculatus	skin	control	SRR22671573	GKYY00000000
		infected	SRR22671571	
	spleen	control	SRR22671574	
		infected	SRR22671572	
Desmognathus conanti	skin	control	SRR22671586	GKYZ00000000
		infected	SRR22671570	
	spleen	control	SRR22671585	
		infected	SRR22671569	
Eurycea cirrigera	skin	control	SRR22671582	GKZA00000000
		infected	SRR22671581	
	spleen	control	SRR22671583	
		infected	SRR22671584	
Eurycea guttolineata	skin	control	SRR22671580	GKZB00000000
		infected	SRR22671578	
	spleen	control	SRR22671579	
		infected	SRR22671577	

Table S2. Host species susceptibility. Category based on mortality during infection trial, if mortality events are recorded the species is considered susceptible

Species	Family	Susceptibility to *Batrachochytrium salamandrivorans*
Desmognathus apalachicola	Plethodontidae	Non-susceptible
Desmognathus auriculatus	Plethodontidae	Susceptible
Desmognathus conanti	Plethodontidae	Non-susceptible
Eurycea cirrigera	Plethodontidae	Susceptible
Eurycea guttolineata	Plethodontidae	Non-susceptible
Tylototriton wenxianensis	Salamandridae	Susceptible
Notophthalmus viridescens	Salamandridae	Susceptible

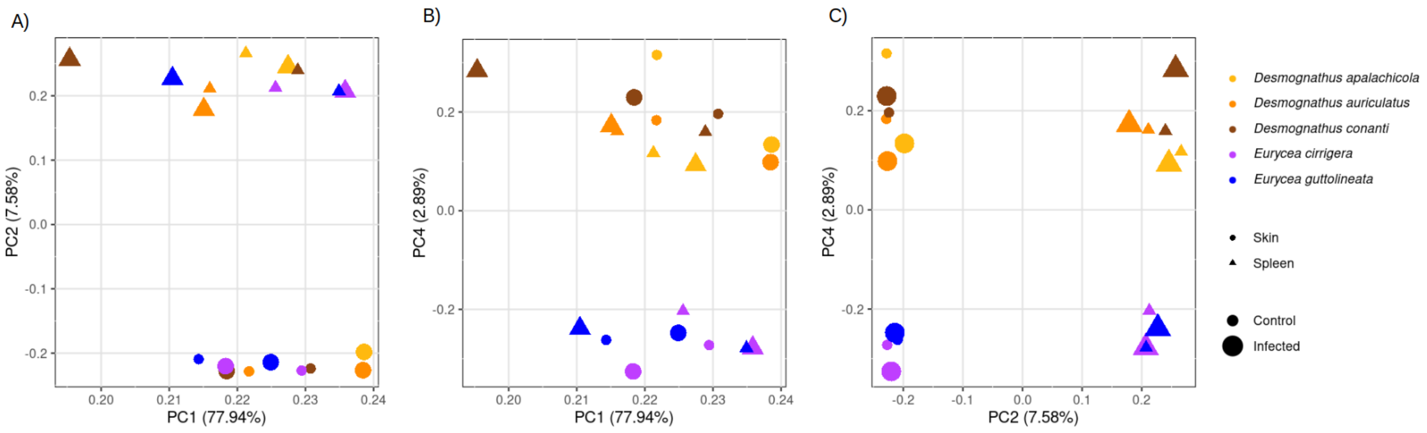

Figure S1. Skin and spleen gene expression of the plethodontid salamander hosts. General gene expression pattern for samples from five hosts (*Desmognathus apalachicola*, *D. auriculatus*, *D. conanti*, *Eurycea cirrigera*, and *E. guttolineata*; represented by color), two tissues (skin and spleen; represented by shape), and different infection categories (control and infected; represented by size). Scatter plots show two components of the principal component analysis (PCA) of gene expression: (A) PC1 versus PC2; (B) PC1 versus PC4; and (C) PC2 versus PC4.
